# Supplementary material for: Detailed molecular and epigenetic characterization of the pig IPEC-J2 and chicken SL-29 cell lines
Source: iScience. 2023 Feb 20;26(3):106252. doi: 10.1016/j.isci.2023.106252 (PMC10018572; doi:10.1016/j.isci.2023.106252)
Supplement: Data S2. Complete homer output for identified motifs in Chicken SL-29, related to Tables 5 and 6 — Homer motif analysis results for histone modifications H3K4me1, H3K4me3, H3K27ac, enhancers, and ATAC-seq of chicken SL-29 cell line. Parameters for possible false positives is as mentioned earlier for S5. [file mmc3.zip › Data_S2/S6/Chicken_SL_29/peak_files_CTCF/homerResults/motif7.similar.html]

motif7

## Information for motif7

A
T
G
C
C
T
A
G
T
A
G
C
C
G
T
A
C
G
A
T
A
C
G
T
G
A
T
C
A
G
T
C
  
Reverse Opposite:  

T
C
A
G
C
T
A
G
C
G
T
A
C
G
T
A
C
G
A
T
A
T
C
G
A
G
T
C
T
A
C
G
  

|  |  |
| --- | --- |
| p-value: | 1e-109 |
| log p-value: | -2.521e+02 |
| Information Content per bp: | 1.717 |
| Number of Target Sequences with motif | 2817.0 |
| Percentage of Target Sequences with motif | 9.56% |
| Number of Background Sequences with motif | 1802.8 |
| Percentage of Background Sequences with motif | 6.20% |
| Average Position of motif in Targets | 150.5 +/- 83.0bp |
| Average Position of motif in Background | 152.9 +/- 91.0bp |
| Strand Bias (log2 ratio + to - strand density) | -0.1 |
| Multiplicity (# of sites on avg that occur together) | 1.06 |
| Motif File: | file (matrix) reverse opposite |

### Similar de novo motifs found

|  |  |  |  |  |  |  |  |
| --- | --- | --- | --- | --- | --- | --- | --- |
| Rank | Match Score | Redundant Motif | P-value | log P-value | % of Targets | % of Background | Motif file |
| 1 | 0.709 | G A C T T C G A T A G C T C A G G T C A T G C A G A C T T A C G G A T C T A C G | 1e-56 | -129.085693 | 9.17% | 6.74% | motif file (matrix) |
| 2 | 0.736 | C T G A A G T C C G T A A C G T A C G T A G T C A C G T A C G T | 1e-24 | -56.742194 | 3.09% | 2.16% | motif file (matrix) |
| 3 | 0.615 | C G T A A G T C C G T A C G T A A C G T A G T C A G T C C G T A C G T A A C T G | 1e-19 | -44.942887 | 0.12% | 0.02% | motif file (matrix) |
